# Sleep-Related Factors and Work-Related Injuries among Farmers in Heilongjiang Province, People’s Republic of China

**DOI:** 10.3390/ijerph110909446

**Published:** 2014-09-11

**Authors:** Huiping Zhu, Yunfeng Han, Yaowu Sun, Zhiping Xie, Xueyan Qian, Lorann Stallones, Huiyun Xiang, Limin Wang

**Affiliations:** 1 Department of Epidemiology and Statistics, Beijing Municipal Key Laboratory of Clinical Epidemiology, School of Public Health, Capital Medical University, Beijing 100069, China; E-Mail: zhuhuiping@ccmu.edu.cn; 2 School of Public Health, Qiqihar Medical University, Qiqihar 161006, China; E-Mails: hyfweb@gmail.com (Y.H.); syw309@126.com (Y.S.); xiezhiping-pp@126.com (Z.X.); xueyanqian@sohu.com (X.Q.); 3 Colorado Injury Control Research Center, Department of Psychology, Colorado State University, Fort Collins, CO 80523, USA; E-Mail: lorann.stallones@colostate.edu; 4 Center for Injury Research and Policy, The Research Institute at Nationwide Children’s Hospital, The Ohio State University College of Medicine, Columbus, OH 43205, USA; 5 Division of Disease Surveillance, National Center for Chronic Disease Control and Prevention, Chinese Center for Disease Control and Prevention, Beijing 100050, China

**Keywords:** sleep-related factors, injury, agriculture, association, Chinese farmers

## Abstract

The association between sleep and work-related injuries among Chinese farmers has not been well studied. This study examined the impact of lack of sleep on agricultural work-related injuries among farmers in China. Data were from a cross-sectional survey of farm-workers in northeastern China. Information was obtained on injuries that occurred in 12 months prior to the survey, on eight sleep-related variables, and on socio-demographic variables. Logistic regression analyses were conducted to test the hypothesis that lack of sleep significantly increased the risk of work-related injuries after controlling for other injury-related risk- factors. Farmers who slept less than six hours per night were 59% more likely to be injured than those who slept more than eight hours per night (OR = 1.59; 95% CI = 1.04, 2.41). The odds of a work-related injury was 2.46 (1.56–3.89) for farmers who reported going to sleep after midnight at least once a week compared with farmers who reported going to sleep after midnight once a month. Farmers who reported having difficulty falling asleep or waking frequently during the night, who often having nightmares, or who experienced daytime sleepiness were at higher injury risk compared with the reference group after controlling for age, gender and alcohol consumption. Reduced sleep hours and poor sleep quality significantly increased the risk of work-related injuries in Chinese farmers. Sleep hours and sleep quality should be considered when assessing occupational safety among farmers.

## 1. Introduction

Agriculture is a major occupation worldwide. It is a physically and emotionally demanding and is related to many health problems, especially work-related injuries [1,2,3,4]. Agriculture is considered one of the most hazardous industries in the United States [5]. Previous research found that in the United States (U.S.) and Canada agriculture continues to be one of the most dangerous occupations [6]. In the United Kingdom (UK), agricultural workers have higher rates of injury than most industries [7]. A recent review of risk factors for agricultural injuries suggested that history of a previous injury, hearing problems, depression, arthritis, and sleep deprivation were significant risk factors for farm injuries [6]. 

Sleep plays a significant role in our overall health. Sleep deprivation as a health issue has received much attention in recent years. Sleep deprivation is defined as not having sufficient sleep, and is generally believed to be associated with a spectrum of adverse health outcomes [8]. Evidence has consistently shown an association between sleep deprivation and hypertension, diabetes mellitus, obesity, breast cancer, and Parkinson’s disease [9,10,11,12,13,14]. Sleep deprivation is a major cause of injuries in transportation and at work [15]. Several studies have addressed the role of sleep deprivation and injury among occupational cohorts and shift workers [16,17]. Leger reported that in the U.S. working population, 52.5% of all work-related injuries were potentially related to sleep deprivation [18]. A study conducted among adolescents living on farms in Colorado found that sleep patterns including oversleeping, falling asleep in afternoon classes, staying up past 3:00 am, and sleeping less than an average of 8.5 h a night were associated with increased risk of injury [19]. 

Insufficient sleep during the night or having a poor quality of sleep may lead to daytime sleepiness that could affect daytime performance and functioning, and impair safety at work [20]. Sleepiness and reduced vigilance have been identified as important factors for risk of traffic and industrial injuries [21]. In a case-crossover study of occupational traumatic hand injuries, workers who slept five or less hours or nine or more hours had an increased risk of injury when compared with workers reporting six to eight hours of sleep per night [22]. Also, a study among veterinarians in Minnesota reported that six or fewer hours of sleep increased the risk of work-related injuries by 80% [23]. 

Few studies have systematically investigated the association between sleep deprivation and work-related injuries among farm workers.. In China, the impact of sleep deprivation on agricultural injuries is not well understood. Our study examined the impact of sleep deprivation on the occurrence of work-related injuries among a sample of farmers in northeastern China. Improved knowledge of sleep deprivation and its potential risk for agricultural work-related injury could provide a modifiable risk factor to target in the development of interventions for the prevention of agricultural injuries in China.

## 2. Methods

### 2.1. Data Source

We analyzed data from a population-based study conducted in 2008 in villages of Qiqihar in the Heilongjiang province, located in northern China. In Qiqihar, approximately 59% of the population lives in agricultural areas. The study methods and procedures were described in detail previously [24]. All study procedures were approved by the Colorado State University Institutional Review Board and the Scientific Research Committee of the Qiqihar Medical University. Informed written consent was obtained from each farmer who participated in our study. 

### 2.2. Study Design

Initially, information about residence was obtained from the local government mandatory registration of residence in Qiqihar. Multistage sampling was used to select the sample for this study. First, nine villages with the same soil type and the same major farming practices were grouped to ensure that our sample represented major agricultural activities in the area. Second, a systematic sampling method was utilized to select 800 families who proportionally represented the number of farmers in each group. Third, we invited rural residents aged 15 years of age and older from the selected families to participate in our survey. 

With help from the Center for Injury Research and Policy (CIRP) of the Research Institute at Nationwide Children’s Hospital and the Colorado Injury Control Research Center (CICRC) at Colorado State University, the research team from the School of Public Health of Qiqihar Medical University developed the questionnaire. The questionnaire was developed using information collected in similar studies conducted by researchers at the Colorado Injury Control Research Center [19]. Our questionnaire considered demographics, history of agricultural works, agriculture work-related injuries, alcohol drinking behaviors, and sleep patterns and sleep hours. The questionnaire was pilot tested among a small group of farmers in the study area. 

In May 2008, 25 post-undergraduate students from the School of Public Health of Qiqihar Medical University trained by the principal investigator (Dr. Limin Wang) conducted face-to-face interviews with farmers. All questionnaires were completed by pairs of the trained interviewers. The interviews were done in either the farmer’s home or at a farm work site. The data were collected from 7 May to 25 May. A research team leader randomly selected 25 questionnaires after completion of the initial survey, and re-interviewed the respondents to check the consistency of the collected data items. This quality check found that more than 90% of survey items were consistently recorded. 

### 2.3. Definition of Injury

Agricultural work-related injuries were defined as injuries that occurred during the previous 12 months to farmers during farm work or farm chores. Respondents were asked to report any injury that resulted in seeking medical attention or in a restriction of normal activities for four hours or more. Injuries that occurred when working in non-farm jobs, taking part in recreational activities, or travelling for non-agricultural work or chores were excluded. Detailed information was collected about the most recent injury. Injury severity was self-reported using the following three categories: mild, moderate and severe. Mild injuries were those treated in an out-patient department, or urgent care center, or by self- or other non-medical people. Hospitalized injuries were defined as moderate. Severe injuries were those resulting in a disability.

### 2.4. Sleep-Related Variables

Eight variables were used to assess sleep patterns average number of hours slept, difficulty to falling asleep at night, difficulty going back to sleep after waking during the night, frequent nightmares, daytime sleepiness, use of sleeping pills, going to sleep after midnight, and self-report of sleep status. Having difficulty falling asleep at night was defined as needing at least 30 min to fall asleep after going to bed. These sleep hours and quality measurements have been used previously by researchers at the Colorado Injury Control Research Center [19]. Because no study has been published about sleep patterns among farmers in China, questions were developed using variables from previous research conducted in the U.S.. 

### 2.5. Statistical Analysis

EpiData 3.02 was used to collect and store the data. Data analyses were conducted using SAS 9.4 statistical software (SAS Institute Inc., Cary, NC, USA). We first compared the percentage of self-reported sleep by age, gender, ethnic group, marital status, and education using Chi-square tests. Second, we calculated the 12-month prevalence of agricultural work-related injuries by demographic characteristics. Chi-square tests were used to compare the prevalence of injury between groups. Third, we analyzed the relationship between sleep-related variables and injuries, and the relationship between sleep-related variables and injury severity by chi-square tests. Finally, to adjust for potential multicollinearity, eight logistic regression models were fitted separately with each sleep-related variable as the main explanatory variable, and injury as the outcome variable after controlling for the potential confounding factors of age, gender and alcohol use.

## 3. Results

A total of 2,264 farmers were initially selected for the study. Of them, 15 refused to participate in the survey and 51 provided incomplete information. A total of 148 people were excluded because they actually spent little time doing farm work. The total sample size for the final statistical analysis in this study was 2,050 farmers. 

According to Table 1, the prevalence of agricultural work-related injuries among farmers was 12.2%. Male farmers had a significantly higher prevalence of injuries than female farmers (14.0% *vs.* 10.4%, *p* = 0.01). Farmers aged 45- to 54-years of age had the highest prevalence of agricultural injuries (16.3%), followed by those aged 25- to 34-years of age. Injury prevalence did not differ significantly among farmers with by ethnicity, marital status and education level (*p* > 0.05).

**Table 1:** Prevalence of agricultural work-related injuries among farmers in a northeastern province, China.

Variables	N	Injured (n)	Injured (%)	χ^2^	*p*
Total	2050	251	12.2		
**Age (year)**				21.2	0.001
15–24	208	17	8.2		
25–34	438	63	14.4		
35–44	516	65	12.6		
45–54	429	70	16.3		
55–64	325	29	8.9		
≥65	134	7	5.2		
**Gender**				6.1	0.01
Male	1075	150	14.0		
Female	975	101	10.4		
**Ethnic group**				1.1	0.58
Han	952	124	13.0		
Daur	1027	118	11.5		
Other	71	9	12.7		
**Marital status**				0.1	0.98
Divorced or widowed	102	13	12.7		
Married	1788	219	12.2		
Never married	160	19	11.9		
**Education**				0.9	0.63
<6 years	828	96	11.6		
7–9 years	1051	131	12.5		
≥10 years	170	24	14.1		

There were 395 (19.3%) farmers who reported sometimes having trouble falling asleep at night, and 184 (9.0%) who reported usually having difficulty falling asleep. Across age groups, the highest percentage of having difficulty falling asleep at night was among farmers aged 55 to 64 years of age (42.2%), followed by those aged 65 years and older (36.8%). Compared with male farmers, female farmers were more likely to have difficulty falling asleep at night. Farmers who were unmarried and farmers whose education was less than 6 years were more likely to have difficulty falling asleep at night compared with others (Table 2).

**Table 2:** Self-reported difficulty falling asleep at night by farmers in a northeastern province, China.

Variables	Never/Rarely	%	Sometimes	%	Often	%	χ^2^	*p*
**Total**	1471	71.8	395	19.3	184	9.0		
**Age (year)**							88.8	<0.001
15–24	172	82.7	29	13.9	7	3.4		
25–34	358	81.7	63	14.4	17	3.9		
35–44	387	75.0	90	17.4	39	7.6		
45–54	280	65.3	96	22.4	53	12.4		
55–64	188	57.8	87	26.8	50	15.4		
>65	86	64.2	30	22.4	18	13.4		
**Gender**							28.8	<0.001
Male	816	75.9	194	18.0	65	6.0		
Female	655	67.2	201	20.6	119	12.2		
**Ethnic group**							1.5	0.82
Han	680	71.4	184	19.3	88	9.2		
Daur	736	71.7	201	19.6	90	8.8		
Other	56	78.9	10	14.1	6	8.5		
**Marital status**							35.6	<0.001
Divorced or widowed	56	54.9	23	22.5	23	22.5		
Married	1290	72.1	340	19.1	158	8.8		
Never married	125	78.1	32	20.0	3	1.9		
**Education**							30.1	<0.001
<6 years	533	64.4	197	23.8	98	11.8		
7–9 years	809	77.0	170	16.1	73	6.9		
≥10 years	129	75.9	28	16.5	13	7.6		

Table 3 presents the agricultural injury prevalence among farmers by sleep-related variables. In this study, 304 farmers (14.5%) reported getting less than six hours of sleep per night, and 48 farmers reported using sleeping pills sometimes or often. Having difficulty falling asleep at night, having difficulty falling back to sleep after waking at night, frequent nightmares, daytime sleepiness, going to sleep after midnight and inadequate sleep by self-report were significantly associated with risk of agricultural injuries. However, neither sleep hours nor sleeping pill use were statistically significantly associated with agricultural injury (*p* > 0.05). Table 4 shows the distribution of severity of agricultural work-related injuries among farmers by sleep-related variables. These results suggested that no sleep-related variables were significantly associated with severity of agricultural injuries. 

**Table 3:** Association between sleep-related variables and agricultural work-related injuries among farmers in a northeastern province, China.

Variables	N	Injured (n)	Injured (%)	χ^2^	*p*
**Sleep hours**				2.5	0.49
<6	304	44	14.5		
6–8	1062	132	12.4		
>8	684	75	11.0		
**Having difficulty falling asleep at night**				10.3	0.01
Never/rarely	1471	160	10.9		
Sometimes	395	58	14.7		
Often	184	33	17.9		
**Having difficulty falling asleep after waking at night**				13.4	0.001
Never/rarely	1475	162	11.0		
Sometimes	380	50	13.2		
Often	195	39	20.0		
**Nightmare frequency**				11.8	0.003
Never/rarely	1415	154	10.9		
Sometimes	525	74	14.1		
Often	110	23	20.9		
**Daytime sleepiness**				15.2	<0.001
Never/rarely	1049	104	9.9		
Sometimes	753	101	13.4		
Often	248	46	18.5		
**Using sleeping pills **				1.9	0.16
Never/rarely	2002	242	12.1		
Sometimes/often	48	9	18.9		
**Going to sleep after midnight**					
Once a month	1736	199	11.5	11.8	0.003
2 to 3 nights per month	179	23	12.8		
At least once a week	135	29	21.5		
**Having adequate sleep by self-report**				9.2	0.01
Often	928	98	10.6		
Sometimes	708	85	12.0		
Never/rarely	414	68	16.4		

**Table 4:** Association between sleep-related variables and severity of agricultural work-related injuries among farmers in a northeastern province, China.

Variables	Mild n (%)	Moderate n (%)	Severe n (%)	χ^2^	*p*
**Total**	137	75	39		
**Sleep hours**				1.4	0.85
>8	43(0.3)	21(0.3)	11(0.3)		
6–8	71(0.5)	42(0.6)	19(0.5)		
<6	23(0.2)	12(0.1)	9(0.2)		
**Having difficulty falling asleep at night**				8.8	0.07
Never/rarely	97(0.7)	44(0.6)	19(0.5)		
Sometimes	26(0.2)	21(0.3)	11(0.3)		
Often	14(0.1)	10(0.1)	9(0.2)		
**Having difficulty falling asleep after waking at night**				3.2	0.53
Never/rarely	94(0.7)	44(0.6)	24(0.6)		
Sometimes	26(0.2)	17(0.2)	7(0.2)		
Often	17(0.1)	14(0.2)	8(0.2)		
**Nightmare frequency**				3.5	0.48
Never/rarely	87(0.6)	46(0.6)	21(0.5)		
Sometimes	35(0.3)	24(0.3)	15(0.4)		
Often	15(0.1)	5(0.1)	3(0.1)		
**Daytime sleepiness**				0.9	0.92
Never/rarely	56(0.4)	33(0.4)	15(0.4)		
Sometimes	57(0.4)	29(0.4)	15(0.4)		
Often	24(0.2)	13(0.2)	9(0.2)		
**Using sleeping pills**				1.1	0.56
Never/rarely	133(0.9)	73(0.9)	36(0.9)		
Sometimes/often	4(0.1)	2(0.1)	3(0.1)		
**Going to sleep after midnight**				1.4	0.85
Once a month	122(0.8)	64(0.8)	36(0.9)		
2 to 3 nights per month	9(0.1)	7(0.1)	2(0.1)		
At least once a week	6(0.1)	4(0.1)	1(0.1)		
**Having adequate sleep by self-report**				8.2	0.09
Often	36(0.2)	19(0.2)	13(0.3)		
Sometimes	39(0.3)	34(0.5)	12(0.3)		
Never/rarely	62(0.5)	22(0.3)	14(0.4)		

Table 5 presents results of eight multivariate logistic regression models. After controlling forage, gender, and alcohol consumption, the OR for agricultural work-related injuries was 1.94 (95% CI: 0.90–4.15) for farmers who used sleeping pills sometimes/often compared with those who never/rarely used sleeping pills. For the rest of models, there was a “dose-response” relationship between sleep-related variables and agricultural work-related injuries. For farmers who reported sleeping fewer than six hours per night, having difficulty falling asleep at night, having difficulty falling asleep after waking at night, having nightmares often, or often experiencing daytime sleepiness were at significantly higher injury risk compared with the reference groups after controlling for age, gender, and alcohol consumption. Farmers who reported going to sleep after midnight at least once a week were over two times more likely to have injuries compared with those who reported going to sleep after midnight once a month.

## 4. Discussion

The present study suggests that sleep disturbances play an important role in the safety of farm workers, as an increased risk of agricultural work-related injuries was found with any decrease in sleep hours, and with increased levels of daytime sleepiness. Additionally, having difficulty falling asleep, sleeping poorly at night, frequent nightmares, difficulty falling asleep after waking up during the night, and inadequate sleep by self-report were significantly associated with the occurrence of agricultural work-related injuries. 

**Table 5:** Eight logistic regression analyses of association between each sleep-related variable and agricultural work-related injury in farmers in China.

Variables	OR ^a^	OR 95% CI
**Sleep hours**		
>8	1	
6–8	1.14	0.83–1.55
<6	1.59	1.04–2.41
**Having difficulty falling asleep at night**		
Never/rarely	1	
Sometimes	1.64	1.17–2.30
Often	2.11	1.36–3.27
**Having difficulty falling asleep after waking at night**		
Never/rarely	1	
Sometimes	1.28	0.90–1.82
Often	2.33	1.55–3.52
**Nightmare frequency**		
Never/rarely	1	
Sometimes	1.40	1.03–1.91
Often	2.37	1.42–2.97
**Daytime sleepiness**		
Never/rarely	1	
Sometimes	1.33	0.99–1.79
Often	2.00	1.35–2.97
**Using sleeping pills**		
Never/rarely	1	
Sometimes/often	1.94	0.90–4.15
**Going to sleep after midnight**		
Once a month	1	
2 to 3 nights per month	0.98	0.61–1.57
At least once a week	2.46	1.56–3.89
**Having adequate sleep by self-report**		
Often	1	
Sometimes	1.17	0.85–1.60
Never/rarely	1.61	1.14–2.27

Note: **^a^** Adjusted OR, controlling for age, gender and alcohol consumption.

In this study, after controlling for potential confounders, farmers who reported sleeping an average of six to eight hours a night had an increased risk of injury (OR = 1.14; 95% CI = 0.83, 1.55). Risk of injury increased for farmers who slept less than six hours a night (OR = 1.59; 95% CI = 1.04, 2.41). Results from a prospective study indicated that sleeping less than 7.5 h per night increased the risk of injury by 61 percent among rural adults in Iowa compared with people who slept longer [25]. Also, a study conducted among rural Minnesota adolescents showed that students who reported an insufficient amount of sleep (6 h or less) every night had an increased risk of injury compared with those who slept nine or more hours every night [26]. Our findings are therefore consistent with the previous studies that suggest duration of sleep is a risk factor for injuries [25,26].

Results from our study also indicated that farmers differed in terms of having difficulty falling asleep by age, which is consistent with previous reports [27,28,29]. Sleepiness has been identified as a risk factor for injuries related to driving [30,31]. Consistent with the studies of driving related injuries [30,31], we found that daytime sleepiness was a risk factor in agricultural work-related injuries. Farmers who reported going to sleep after midnight at least once a week were over two times more likely to have injuries comparing with those who go to sleep after midnight once a month in our study. This finding is consistent with results from previous studies among adolescents indicating that time one goes to sleep has an impact on the risk of injury [32,33,34,35]. The time it takes to fall asleep may play a role in injury risk in both direct and indirect ways. The direct impact may be related to the increased number of hours awake, which thus increase risk of exposure. Indirectly, a falling asleep late might lead to fewer total hours of sleep which has been associated with risk of injuries Giannotti *et al.* found that adolescents in Italy who chose to go to sleep later reported more frequent attention problems, more daytime sleepiness, greater emotional problems, higher use of sleeping aids and caffeine, and more injuries compared with their peers [29].

Gender may play a unique role in the associations between sleep and injuries. A study from Italy reported girls were more likely than boys to report poor sleep quality including long periods of being awake at night and waking up early [29]. Additional studies have reported females have a range of sleep problems more often than males [36,37,38,39]. Similar to the findings from these previous studies, our study found that female farmers reported having difficulty falling asleep at night more often than male famers.

Difficulty falling asleep is a subtype of insomnia. Nakata *et al.* reported that insomnia symptoms and difficulty falling asleep were associated with occupational injuries [40]. Leger *et al.* found that compared with good sleepers, individuals with severe insomnia had more problems at work including decreased concentration, difficulty performing duties and more work-related injuries [41]. In addition, workers who had sleep disorders in the construction industry reported having occupational injuries more often than those without sleep disorder, and the injuries that occurred tended to be serious [42]. Balter and Ulenhuth found that the annual rate of serious injuries in patients with chronic untreated insomnia were 4.5 times higher than the annual injury rate in normal controls [43]. Our results are consistent with the research on insomnia symptoms as risk factors for the occurrence of injuries.

We observed a strong association between awakening sleep at night and between difficulty falling asleep after waking up at night and the occurrence of work-related injuries. Lavie *et al.* also reported that frequent mid-sleep awakenings were more strongly associated with the incidence of injuries than difficulty falling asleep [44]. The difference between results between the two studies might be related to the use of different definitions for difficulty falling asleep. Lavie *et al.* used a more strict definition of 45 min or more to fall asleep while we used 30 minutes or more. Others have reported that difficulty in sleep among workers was associated with fatal injuries [45]. Thus, workers with poor sleep patterns should be especially cautious when involved in dangerous work, such as agricultural work.

The current study had several limitations. . First, this study was a cross-sectional design, which made it impossible to identify the direction of the causal relationships. Second, sleep patterns were based on self-report. In addition, the amount of sleep per night was collected to obtain an estimated average rather than exact sleep hours prior to an injury. Third, not all relevant factors which may have an impact on both sleep and injury were measured in our study. For example, working hours, psychosocial and physical/psychological conditions were not assessed in our study. Previous studies have suggested that recall bias may result in inaccurate reports of injuries. Further inconsistent recall of sleep hours and sleep patterns may occur in surveys based on self- reporting [46,47]. 

## 5. Conclusions

Reduced sleep hours and quality may increase the risk of agricultural work-related injuries in farmers in China. Findings of this study underscore an important occupational safety issue facing millions of farmers in China. Future study needs to be done using objective measures sleep hours and quality to verify the association between lack of sleep and risk of agricultural injuries in China.
